# Cross-sectional and longitudinal associations between pan-immune-inflammation value and serum uric acid

**DOI:** 10.3389/fendo.2025.1720450

**Published:** 2025-12-10

**Authors:** Zi-Long Lu, Rui Chu, Ling-Meng Wang, Yi Cheng, Jin-Yi Gong, Yong-Yan Huo, Chang-Sheng Sheng

**Affiliations:** 1 Department of Cardiovascular Medicine, Center for Epidemiological Studies and Clinical Trials and Center for Vascular Evaluation, Shanghai Key Lab of Hypertension, Shanghai Institute of Hypertension, Ruijin Hospital, Shanghai Jiaotong University School of Medicine, Shanghai, China; 2 Department of General Practice, Waigang Community Health Service Center of Jiading District, Shanghai, China

**Keywords:** serum uric acid, hyperuricemia, pan-immune inflammation value, inflammation, elderly

## Abstract

**Background:**

Elevated serum uric acid (SUA) correlates with inflammation, but the pan-immune inflammation value (PIV)—a novel integrated inflammatory marker—has not been explored in relation to SUA. We investigated cross-sectional and longitudinal PIV-SUA associations.

**Methods:**

We analyzed 5,766 participants aged ≥60 years from a 2018 cardiovascular examination cohort with 2022 follow-up. The PIV was calculated as neutrophil number × platelet number × monocyte number/lymphocyte number, with cell counts expressed as ×1000 cells/μL. Hyperuricemia was defined as SUA concentrations ≥ 420 μmol/L (7 mg/dL) in males and ≥ 360 μmol/L (6 mg/dL) in females. Cross-sectional associations were assessed via multivariate linear/logistic regression; longitudinal associations via Cox regression.

**Results:**

At baseline, hyperuricemia prevalence was 22.4% among 5,766 participants (mean age 68.5 years). Restricted cubic spline showed a nonlinear PIV-SUA relationship. In fully adjusted models, each 1-SD PIV increase associated with higher SUA (β ± SE: 3.7 ± 1.1; P<0.0001). PIV quartiles (vs. lowest) showed β values: Q2 = 7.5, Q3 = 6.7, Q4 = 12.5 (P trend<0.001). Logistical regression revealed each 1-SD PIV increase linked to higher hyperuricemia risk (OR = 1.12, 95%CI 1.05-1.29; P = 0.0003). PIV quartiles (vs. lowest) had ORs: Q2 = 1.27, Q3 = 1.23, Q4 = 1.54 (P trend<0.001). Over 4-year follow-up, Cox regression indicated a J-curve relationship between PIV and SUA/hyperuricemia, with the lowest risk at PIV quartile 2.

**Conclusions:**

PIV showed a nonlinear relationship with serum uric acid and hyperuricemia in cross-sectional analyses, while exhibiting a J-curve relationship in longitudinal studies. These suggest dynamic interactions between inflammatory markers and uric acid metabolism, dependent on inflammation duration.

## Introduction

Uric acid, the end metabolic product of purine nucleotides, plays an important role in cellular homeostasis. It is biosynthesized endogenously through energy metabolism pathways. Hyperuricemia demonstrates strong associations with obesity, hypertension, diabetes, dyslipidemia, and chronic kidney disease (1, 2). Epidemiological studies show that hyperuricemia prevalence is rising globally. A Chinese survey revealed that hyperuricemia prevalence among Chinese adults rose from 11.1% (95% confidence interval: 10.3%-11.8%) in 2015–16 to 14.0% (13.1%-14.8%) in 2018-19, showing a significant upward trend (3).

Systemic inflammation is fundamental to many chronic diseases, affecting cardiovascular, renal, and metabolic systems. The Pan-Immune Inflammation Value (PIV) provides a comprehensive assessment of systemic immune inflammatory responses by integrating key peripheral blood immune cells: neutrophils, platelets, monocytes, and lymphocytes. Inflammatory factors promote atherosclerosis, endothelial dysfunction, and vascular stiffness, leading to hypertension and heart failure. Moreover, inflammation impairs kidney function through glomerular injury and fibrosis, accelerating chronic kidney disease (CKD) progression. It is also closely linked to insulin resistance, a hallmark of type 2 diabetes and metabolic syndrome. Thus, inflammation plays a crucial role in cardiovascular disease development and progression (4–6).

Inflammation is central to hyperuricemia pathogenesis. Research has confirmed that various inflammatory biomarkers—including high-sensitivity C-reactive protein (hs-CRP), systemic inflammatory response index (SIRI), and monocyte to high-density lipoprotein cholesterol ratio (MHR)—positively correlate with hyperuricemia (7–9). The Systemic Immune-inflammation Index (SII) also shows association with elevated uric acid (10). Furthermore, uric acid promotes inflammation by activating pro-inflammatory cytokines and increasing oxidative stress, creating a self-reinforcing cycle (11). A previous study revealed that PIV was superior to the neutrophil-lymphocyte ratio, platelet-lymphocyte ratio, and systemic immune-inflammation index for predicting primary and secondary outcomes in ST-segment elevation myocardial infarction (STEMI) (4). Another study showed that PIV demonstrated a promising association with poor overall survival and progression-free survival compared with other biomarkers in Patients with Metastatic Melanoma (12). However, the relationship between PIV, a novel and promising inflammatory indicator, and serum uric acid (SUA) remains unexplored.

Therefore, this study aims to investigate the association between PIV and SUA using health examination data from elderly Shanghai residents. Through a 4-year follow-up, we will examine their prospective relationship, providing evidence for early prevention of elevated blood uric acid.

## Methods

Our study was conducted in the framework of the Chronic Disease Detection and Management in the Elderly (≥60 years) Program supported by the municipal government of Shanghai. In an urbanized suburban town, 40 kilometers from the city center, we invited all residents of 60 years or older to take part in comprehensive examinations of cardiovascular disease and risk annually from 2018 to 2022. The Ethics Committee of Ruijin Hospital, Shanghai Jiaotong University School of Medicine approved the study protocol. All subjects gave written informed consent.

In 2018, 6,678 elderly participants (≥60 years) underwent comprehensive examinations, and in 2022, 6,812 elderly participants (≥60 years) underwent these examinations. Of these, 5,794 subjects participated in both examination periods. We excluded 28 subjects from the present analysis because of missing important information. Thus, the number of participants included in the present analysis was 5766.

### Field work

One experienced physician administered a standardized questionnaire to collect information on medical history, lifestyle and use of medications. A trained technician measured body height and body weight. Body mass index was calculated as the body weight in kilograms divided by the body height in meters squared. Blood pressure was measured with a validated blood pressure monitor (Omron, Inc., Tokyo, Japan) (13). Hypertension was defined as a blood pressure of at least 140 mmHg systolic or 90 mmHg diastolic or as the use of antihypertensive drugs (14).

### Measurements

Venous blood samples were drawn after overnight fasting for the measurement of blood routine, plasma glucose and serum uric acid and lipids. The pan-immune-inflammation value is calculated by neutrophil number × platelet number × monocyte number/lymphocyte number, with cell counts represented as ×1000 cells/uL (15). Diabetes mellitus was defined as a plasma glucose concentration of at least 7.0 mmol/L fasting or 11.1 mmol/L at any time or as the use of anti-diabetic agents (16). Hyperuricemia was diagnosed when serum uric acid concentrations were ≥ 420 μmol/L (7 mg/dL) in males and ≥ 360 μmol/L (6 mg/dL) in females (17).

### Statistics analysis

For database management and statistical analysis, we used SAS software (version 9.4, SAS Institute, Cary, NC, USA). Means and proportions were compared with the standard normal z-test and Fisher’s exact test, respectively. We conducted a study combining cross-sectional analysis (2018 data) and longitudinal analysis (using 2018 PIV to predict 2022 outcomes) within a community-based cohort. We explicitly state that baseline (2018) PIV was used to predict the follow-up (2022) outcomes (change in SUA or incidence of hyperuricemia), controlling for baseline covariate. We performed multiple linear regression analysis or logistic regression analyses to study the association between serum uric acid or hyperuricemia and PIV, while controlling for covariates. In the partially adjusted analysis (Model 1), we adjusted for age and sex. In the fully adjusted models (Model 2), we adjusted for age, sex, body mass index, total cholesterol, serum triglycerides, fasting glucose, and systolic and diastolic blood pressure. We further classified PIV into quartiles, treating quartile 1 as the reference in the cross-sectional analysis and quartile 2 as the reference in the longitudinal analysis.

## Results

### Baseline characteristics of the study participants

The study included 5766 participants with a mean age of 68.5 ± 6.2 years. Of those, 2521 (43.7%) were men and 3245 (56.3%) were women. The mean serum uric acid level was 331.1 ± 87.8 μmol/L, with 22.4% (1,291) of participants diagnosed with hyperuricemia. Blood cell counts per 10^9^/L averaged 3.37 ± 1.10 for neutrophils, 1.79 ± 0.54 for lymphocytes, 0.35 ± 0.13 for monocytes, and 201 ± 58 for platelets. The mean PIV was 147.3 ± 115.4 (**Table 1**).

**Table 1:** Characteristics of study population by quartiles of pan immunity inflammation value (PIV).

Characteristics	Overall (n=5766)	Quartiles of PIV				*P* value
		Q1 (n=1439)	Q2 (n=1447)	Q3 (n=1438)	Q4 (n=1442)	
Age (years)	68.5± 6.2	68.3± 6.1	68.3± 6.1	68.5± 6.1	68.9± 6.3	0.07
Men, n (%)	2521 (43.7)	458 (31.8)	574 (39.7)	670 (46.6)	819 (56.8)	<0.0001
BMI, Kg/m^2^	24.9± 3.2	24.4± 3.2	25.0± 3.2	25.1± 3.1	25.0± 3.1	<0.0001
SBP, mmHg	133.5± 19.8	132.2± 18.1	132.9± 18.0	134.5± 24.0	134.5± 18.6	0.002
DBP, mmHg	74.4± 10.2	73.6± 10.0	74.1± 10.2	74.8± 10.3	75.3± 10.1	<0.0001
Total Cholesterol, mmol/L	4.96± 0.96	4.97± 0.95	4.98± 0.92	4.93± 0.96	4.95± 1.01	0.58
Triglycemia, mmol/L	1.64± 1.04	1.53± 1.10	1.62± 0.99	1.71± 1.17	1.69± 0.88	<0.0001
HDL_c, mmol/L	1.34± 0.31	1.39± 0.32	1.37± 0.33	1.31± 0.29	1.30± 0.31	<0.0001
LDL_c, mmol/L	3.04± 0.85	3.03± 0.84	3.04± 0.83	3.04± 0.85	3.07± 0.87	0.57
Serum Creatinine, mmol/L	70.6± 16.6	67.4± 14.3	69.6± 15.8	71.6± 16.9	73.6± 18.5	<0.0001
Serum uric acid, μmmol/L	331.1± 87.8	313.4± 81.5	329.7± 89.2	335.0± 85.2	346.1± 91.7	<0.0001
Fasting Glucose, mmol/L	5.85± 1.82	5.74± 1.52	5.86± 1.80	5.90± 1.90	5.91± 2.00	0.04
Hba1c, %	5.94± 1.00	5.79± 0.86	5.94± 1.02	6.00± 1.06	6.04± 1.03	<0.0001
Neutrophil, 10^9^/L	3.37± 1.10	2.45± 0.60	3.01± 0.64	3.51± 0.68	4.51± 1.15	<0.0001
Lymphocyte, 10^9^/L	1.79± 0.54	1.74± 0.54	1.80± 0.51	1.82± 0.54	1.79± 0.56	0.0003
Monocyte, 10^9^/L	0.35± 0.13	0.25± 0.08	0.31± 0.08	0.37± 0.09	0.47± 0.13	<0.0001
Platelet, 10^9^/L	201± 58	161± 47	191 ± 46	210± 48	242± 58	<0.0001
PIV	147.3± 115.4	55.1± 15.1	96.6± 11.5	145.6± 18.2	291.8± 143.7	<0.0001
Hyperuricemia, n (%)*	1291 (22.4)	252 (17.5)	327 (22.6)	328 (22.8)	384 (26.6)	<0.0001

Authors’ analysis of the study cohort data. Values are mean± SD or number of subjects (%). BMI, Body mass index; SBP, systolic blood pressure; DBP, diastolic blood pressure; HDL, high density lipoprotein; LDL, Ligh density lipoprotein; Hba1C, hemoglobin A1c. *For the definition of hyperuricemia, see Methods section.

We divided PIV into quartiles: Q1 (n=1439, mean 55.1), Q2 (n=1447, mean 96.6), Q3 (n=1438, mean 145.6), and Q4 (n=1442, mean 291.8). Serum uric acid concentration demonstrated a significant upward trend with increasing PIV quartiles, from 313.4 ± 81.5 μmol/L in Q1 to 346.1 ± 91.7 μmol/L in Q4. Hyperuricemia prevalence showed a corresponding increase across these quartiles, from 17.5% in Q1 to 26.6% in Q4 (**Table 1**).

### Cross-sectional associations between PIV and serum uric acid or hyperuricemia

**Figure 1:**
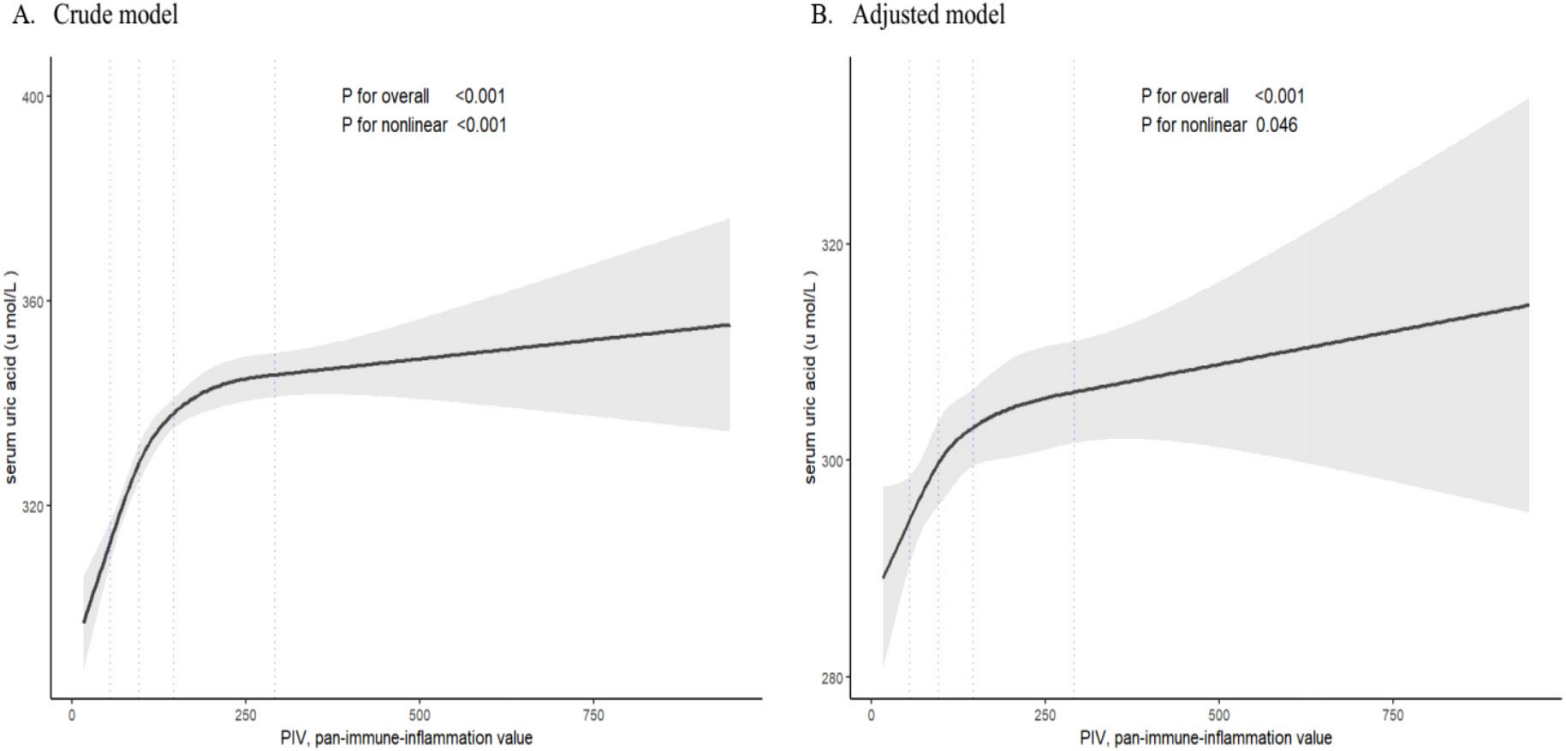
Restricted cubic splines of the associations between pan-immune-inflammation value (PIV) and serum uric acid (SUA) in crude and adjusted model. Authors’ analysis of the study cohort data.

**Figure 2:**
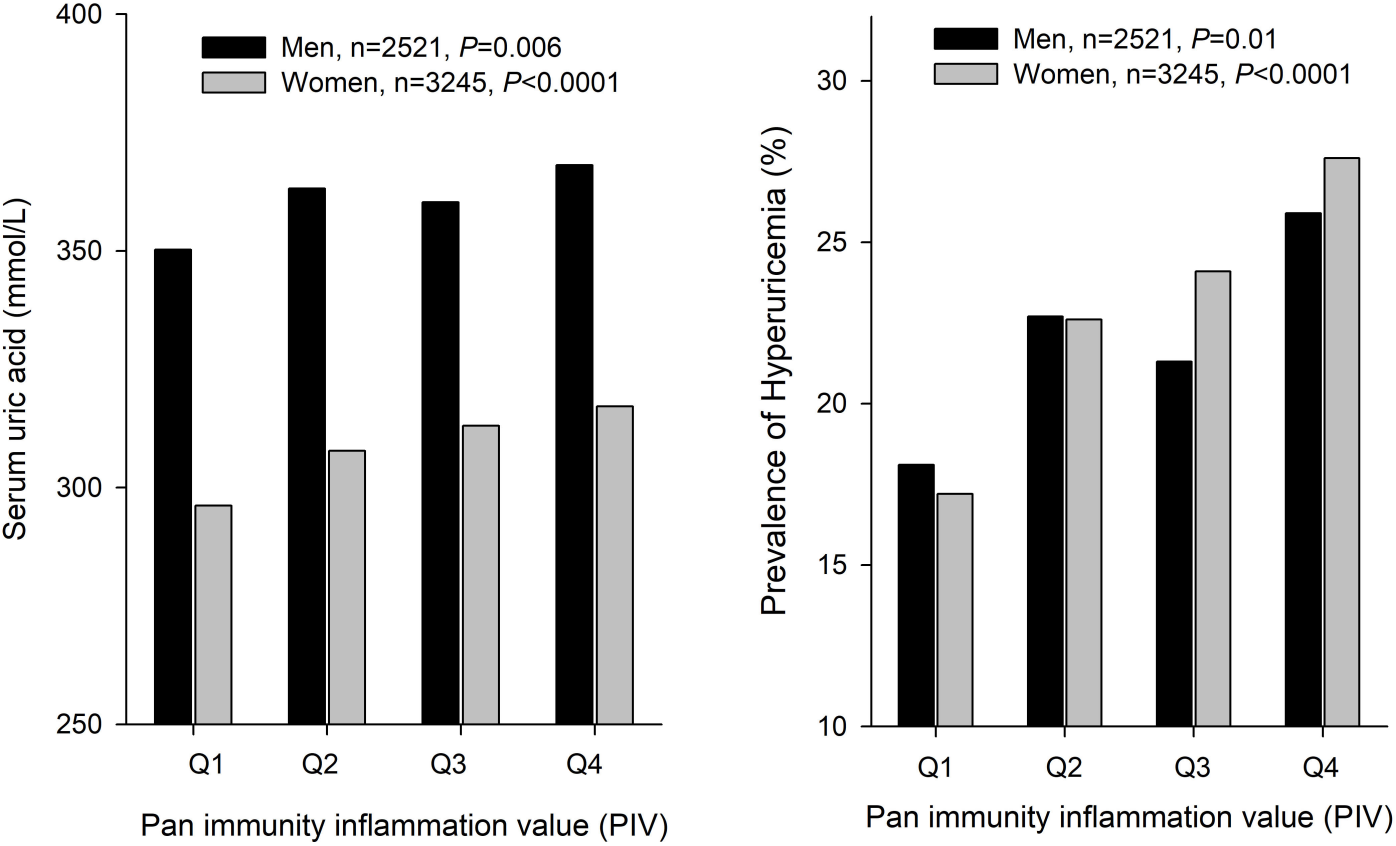
Associations between Pan-Immune-Inflammation Value (PIV) quartiles and serum uric acid (SUA, Left) or hyperuricemia (Right). Authors’ analysis of the study cohort data.

**Table 2** displays the cross-sectional association between PIV and serum uric acid or hyperuricemia at baseline. A 1-SD increase in PIV was associated with increased serum uric acid in the crude model (β ± SE, 9.4 ± 1.1; *P* < 0.0001), partially adjusted model (β ± SE, 5.1 ± 1.1; *P* < 0.0001), and fully adjusted model (β ± SE, 3.7 ± 1.1; *P* < 0.0001). After full adjustment, the prevalence of hyperuricemia increased by 12% (OR [95%CI], 1.12 [1.05-1.29]; *P* = 0.0003) with each 1-SD increase in PIV. To explore the relationship between PIV and serum uric acid, we employed a restricted cubic spline (**Figure 1**). The relationships between PIV and serum uric acid exhibited nonlinear patterns in both unadjusted and adjusted models. **Figure 2** illustrates the relationships between PIV quartiles and serum uric acid concentrations or hyperuricemia prevalence by gender. Both serum uric acid concentrations and hyperuricemia prevalence increased progressively with higher PIV quartiles in men and women.

**Table 2:** Cross-sectional association between pan immunity inflammation value (PIV) and serum uric acid or hyperuricemia.

PIV	Serum uric acid, +1 μmol/L		Hyperuricemia	
	β ± SE	*P*	OR (95%CI)	*P*
Crude model				
Continuous PIV (+ 1 SD)	9.4 ± 1.1	<0.0001	1.14 (1.08-1.21)	<0.0001
Categories PIV				
Q1	0 (Ref.)	–	1 (Ref.)	–
Q2	16.3 ± 3.2	<0.0001	1.38 (1.14-1.65)	0.0007
Q3	21.6 ± 3.2	<0.0001	1.39 (1.16-1.67)	0.0004
Q4	32.7 ± 3.2	<0.0001	1.71 (1.43-2.05)	<0.0001
*P* for Trend	<0.0001		<0.0001	
Adjusted model 1				
Continuous PIV (+ 1 SD)	5.1 ± 1.1	<0.0001	1.14 (1.07-1.21)	<0.0001
Categories PIV				
Q1	0 (Ref.)	–	1 (Ref.)	–
Q2	12.3 ± 3.1	<0.0001	1.38 (1.15-1.66)	0.0006
Q3	13.7 ± 3.1	<0.0001	1.39 (1.16-1.68)	0.0004
Q4	19.0 ± 3.1	<0.0001	1.70 (1.42-2.04)	<0.0001
*P* for Trend	<0.0001		<0.0001	
Adjusted model 2				
Continuous PIV (+ 1 SD)	3.7 ± 1.1	0.0005	1.12 (1.05-1.19)	0.0003
Categories PIV				
Q1	0 (Ref.)	–	1 (Ref.)	–
Q2	7.5 ± 2.9	0.01	1.27 (1.05-1.54)	0.01
Q3	6.7 ± 3.0	0.02	1.23 (1.01-1.49)	0.04
Q4	12.5 ± 3.0	<0.0001	1.54 (1.27-1.86)	<0.0001
*P* for Trend	<0.0001		<0.0001	

Authors’ analysis of the study cohort data.

a Crude model, no covariate was adjusted;

b Adjusted model 1, adjusted for age and sex;

c Adjusted model 2, adjusted for age, sex, body mass index, total cholesterol, serum triglycerides, fasting glucose, and systolic and diastolic blood pressure.

Quartile 1 (Q1) of PIV was used as the reference in cross-sectional analysis.

Upon categorizing PIV into quartiles to investigate the association with serum uric acid in the fully adjusted model, the β values for quartiles 2, 3, and 4, relative to the lowest PIV quartile, were 7.5, 6.7, and 12.5, respectively (*P* for trend <0.001). Similar results were observed for PIV quartiles and hyperuricemia in the fully adjusted model, where the OR values for quartiles 2, 3, and 4, relative to the lowest PIV quartile, were 1.27, 1.23, and 1.54, respectively (*P* for trend <0.001). Generally, the PIV showed a nonlinear relationship with serum uric acid and hyperuricemia in cross-sectional association.

### Longitudinal associations between PIV and serum uric acid or hyperuricemia

**Table 3** displays the longitudinal association between PIV and serum uric acid or hyperuricemia during 4-year follow up. Among 3311 participants whose serum uric acid increased from 2018 to 2022, a 1-SD increase in PIV was not significantly associated with increased serum uric acid in the crude model (β ± SE, 1.5 ± 0.9; *P* = 0.09), partially adjusted model (β ± SE, 1.4 ± 0.9; *P* = 0.13), and fully adjusted model (β ± SE, 1.1 ± 0.9; *P* = 0.23). Similarly, among 4475 participants without hyperuricemia in 2018, the incidence of hyperuricemia in 2022 was not associated with 1-SD increases in PIV from 2018 across crude, partially adjusted, or fully adjusted models.

**Table 3:** Longitudinal associations between pan-immune-inflammation value and serum uric acid increases or hyperuricemia incidence.

PIV	Serum uric acid increase		Incidence of Hyperuricemia	
	β ± SE	*P*	HR (95%CI)	*P*
Crude model				
Continuous PIV (n=3311, + 1 SD)	1.5 ± 0.9	0.09	1.02 (0.94-1.10)	0.66
Categories PIV (n=4475)				
Q1	1.16 ± 2.56	0.65	1.12 (0.90-1.39)	0.31
Q2	0 (Ref.)	–	1 (Ref.)	–
Q3	4.28 ± 2.61	0.10	1.14 (0.92-1.42)	0.23
Q4	6.37 ± 2.62	0.01	1.26 (1.01-1.57)	0.04
*P* for Trend	0.02		0.16	
Adjusted model 1				
Continuous PIV (+ 1 SD)	1.4 ± 0.9	0.13	1.02 (0.95-1.11)	0.55
Categories PIV				
Q1	1.46 ± 2.54	0.57	1.10 (0.89-1.38)	0.36
Q2	0 (Ref.)	–	1 (Ref.)	–
Q3	4.20 ± 2.59	0.11	1.16 (0.93-1.44)	0.19
Q4	6.13 ± 2.62	0.02	1.27 (1.02-1.58)	0.03
*P* for Trend	0.03		0.13	
Adjusted model 2				
Continuous PIV (+ 1 SD)	1.1 ± 0.9	0.23	1.00 (0.92-1.09)	0.98
Categories PIV				
Q1	2.40 ± 2.52	0.34	1.20 (0.96-1.49)	0.11
Q2	0 (Ref.)	–	1 (Ref.)	–
Q3	3.20 ± 2.57	0.21	1.12 (0.90-1.40)	0.30
Q4	5.49 ± 2.60	0.03	1.24 (0.99-1.55)	0.06
*P* for Trend	0.14		0.56	

Authors’ analysis of the study cohort data.

a Crude model, no covariate was adjusted;

b Adjusted model 1, adjusted for age and sex;

c Adjusted model 2, adjusted for age, sex, body mass index, total cholesterol, serum triglycerides, fasting glucose, and systolic and diastolic blood pressure.

Quartile 2 (Q2) of PIV was used as the reference in longitudinal analysis.

Upon categorizing PIV into quartiles to investigate the association with serum uric acid in the fully adjusted model, the β values for quartiles 1, 3, and 4, relative to the quartile 2 of PIV, were 2.40, 3.20, and 5.49, respectively. Similar results were observed for PIV quartiles and hyperuricemia in the fully adjusted model, where the HR values for quartiles 1, 3, and 4, relative to the quartile 2 of PIV, were 1.20, 1.12, and 1.24, respectively. Generally, the PIV showed a J-curve relationship with serum uric acid and hyperuricemia in longitudinal association.

Generally, PIV demonstrated a nonlinear relationship with serum uric acid and hyperuricemia in cross-sectional analyses, while showing a J-curve relationship with these parameters in longitudinal associations. We conducted sensitivity analysis to examine the J-shaped curve phenomenon, and found that this pattern remained consistent across different genders (men vs. women), body mass index (BMI) levels (<24 vs. ≥24 kg/m²), and age groups (<80 vs. ≥80 years).

## Discussion

In the present study, we investigated both cross-sectional and longitudinal associations between PIV and serum uric acid. PIV demonstrated a nonlinear relationship with serum uric acid and hyperuricemia in cross-sectional analyses, while showing a J-curve relationship with these parameters in longitudinal associations.

The PIV serves as a novel indicator of systemic inflammation by incorporating neutrophils, platelets, monocytes, and lymphocytes—the primary immune cell types in peripheral blood that reflect systemic inflammatory status (15, 18–20). Recent PIV research has mainly examined its influence on cancer prognosis and treatment outcomes (21, 22). Zhai et al. demonstrated that PIV functions as an independent prognostic indicator for non-small-cell lung cancer (NSCLC) patients achieving pathological complete remission after neoadjuvant immunochemotherapy (23). Additionally, Provenzano et al. conducted a retrospective analysis showing associations between elevated PIV and adverse outcomes, including reduced overall survival (HR: 4.46, 95% CI: 2.22-8.99) and shorter progression-free survival (HR: 2.03, 95% CI: 1.08-3.80) (24). PIV has also shown strong correlations with cardiovascular indicators. In a study of 3,047 participants, researchers identified a significant positive correlation between PIV and abdominal aortic calcification (AAC). After full adjustment, every 100-unit increase in PIV corresponded to a 0.055-point increase in AAC score (β=0.055, 95% confidence interval: 0.014-0.095). When researchers divided PIV into quartiles, they observed a clear pattern: AAC scores increased steadily as PIV quartiles rose (25).

Our findings reveal that elevated PIV independently correlates with higher SUA levels (β=3.71 for 1μmol/L increase, *P* = 0.0005) and increased hyperuricemia risk (OR = 1.12, *P* = 0.0003) in cross-sectional analysis. This finding aligns with previous National Health and Nutrition Examination Survey (NHANES)research. In a cross-sectional analysis of over 30,000 cases, hyperuricemia prevalence progressively increased with elevated PIV levels (12). Participants in the fourth quartile of PIV had a greater risk of hyperuricemia compared to those in the first quartile (OR = 1.19, 95% CI: 1.07–1.32, P = 0.001). Additionally, a NHANES study of over 13,000 cases demonstrated that uric acid mediated the relationship between PIV and Klotho, a marker of aging (26). Longitudinal analysis after 4 years showed that participants in the 4^th^ PIV quartile had significantly higher uric acid levels compared to those in the 2^nd^ quartile (β=5.49, *P* = 0.03), suggesting a potential association. As PIV can be calculated from routine blood count parameters, it offers an economical, non-invasive method for monitoring hyperuricemia risk. This biomarker can guide clinicians in developing targeted strategies for patients with elevated SUA, ultimately enhancing preventive care and treatment outcomes.

Our longitudinal study explored the association between inflammation and hyperuricemia, which showed a distinct J-shaped curve. Interestingly, moderate inflammation (2^nd^ quartile) – not the lowest inflammatory state – correlated with the best outcomes, showing the smallest increase in serum uric acid levels and lowest hyperuricemia incidence after four years of follow-up. This J-shaped pattern suggests complex interactions between inflammatory responses and uric acid metabolism, likely involving multiple regulatory mechanisms and metabolic pathways (27, 28). To our knowledge, no previous studies have found a similar J-shaped phenomenon in longitudinal analysis. Potential explanations for this J-shaped phenomenon include immune dysregulation, subclinical malnutrition, or other unmeasured frailty-related factors that could influence both low-grade inflammation and uric acid metabolism. The specific molecular and cellular mechanisms underlying this non-linear relationship remain unclear, highlighting the need for further basic research to uncover the biological basis of this clinical observation.

The pathophysiological mechanism of the PIV-SUA association remains unclear, but several potential explanations exist. Higher PIV levels reflect either increased neutrophils, monocytes, and platelets or decreased lymphocytes. During inflammation, platelets interact with neutrophils and lymphocytes, promoting monocyte adhesion and transport. This process releases inflammatory factors like TNF-α, IL-6, and IL-1, which may enhance uric acid production by upregulating xanthine oxidase activity (29, 30). Conversely, uric acid itself may trigger systemic inflammation by activating the pro-inflammatory NF-κB signaling pathway (31). Sodium urate crystals can also stimulate neutrophil activation through the NALP3 inflammasome, generating immune mediators that promote inflammation (32). Additionally, neutrophil dysfunction may impair renal clearance of soluble uric acid, leading to free radical production and pro-oxidant effects (33).

PIV integrates four distinct immune cell lineages (neutrophils, platelets, monocytes, lymphocytes) into a single metric, potentially offering a more comprehensive reflection of the systemic immune-inflammatory state than individual cell counts or CRP alone. It might capture different aspects of the immune response (e.g., innate vs. adaptive balance, pro-thrombotic inflammation) and is derived directly from the ubiquitous and inexpensive complete blood count (CBC), requiring no additional testing. Further research is needed to directly compare its predictive performance against established markers like CRP in the context of hyperuricemia.

Our study has several limitations. Despite adjusting for multiple important covariates, we cannot entirely rule out residual confounding factors such as physical activity, dietary habits, genetic background, and other lifestyle variables that influence uric acid metabolism. Specific medications (e.g., diuretics, urate-lowering therapy), detailed dietary purine intake, and alcohol consumption were not systematically available for all participants in this large community-based cohort, which may also affect uric acid metabolism. Furthermore, our study was confined to a suburban Shanghai population, whose specific geographical, lifestyle, and genetic characteristics may limit the generalizability of our findings to other regions or populations. Future validation studies across diverse regions, ethnicities, and populations are necessary.

In conclusion, our study shows that PIV demonstrated a nonlinear relationship with serum uric acid and hyperuricemia in cross-sectional analyses, while exhibiting a J-curve relationship in longitudinal investigations. This indicates that inflammatory marker interactions with uric acid metabolism change dynamically based on inflammation persistence. Our results provide insights for diagnosis, risk assessment, and potential new treatments for hyperuricemia and related conditions, potentially enabling more personalized treatment approaches that account for patients’ inflammatory status.

## Data Availability

The raw data supporting the conclusions of this article will be made available by the authors, without undue reservation.
